# PES Syndrome Presenting as Severe Hyponatremia in an Asymptomatic Septuagenarian

**DOI:** 10.1155/2021/8891881

**Published:** 2021-01-05

**Authors:** Rahul Pansare, Sangeetha Nanthabalan

**Affiliations:** Department of Internal Medicine, St. Mary Mercy Hospital, Livonia, MI 48154, USA

## Abstract

**Background:**

Hyponatremia is commonly seen in hospitalized patients. In euvolemic individuals, syndrome of inappropriate antidiuretic hormone secretion (SIADH) is a common differential. However, before establishing a diagnosis of SIADH, it is imperative to evaluate for hypocortisolism and hypothyroidism. The finding of endocrine abnormalities determined to be of pituitary origin should prompt evaluation by brain MRI. Furthermore, primary empty sella (PES) is commonly seen as an incidental neuroradiological finding. However, PES in association with endocrine abnormalities is recognized as a separate entity called primary empty sella syndrome (PESS). *Case Presentation.* We report the case of a 71-year-old male sans neurological symptoms who presented to us with severe hyponatremia in whom we used a stepwise approach which led us to the diagnosis of PESS. This methodical approach was crucial for timely correction of the endocrine abnormalities which in turn rectified hyponatremia. Intriguingly, the presence of an ectopic pituitary which is a very rare entity and the sudden manifestation of his underlying endocrine deficiencies in the 8th decade of life make this clinical scenario highly unusual.

**Conclusion:**

Clinicians should be aware that absence of an orderly approach to workup presumed SIADH or an assumption of PES (instead of PESS) could both lead to serious consequences in the face of missed endocrine deficiencies.

## 1. Background

Hyponatremia is a common electrolyte derangement observed in hospitalized patients. Severe hyponatremia (<120 mMol/L) has been reported to be a predictor of high mortality [1, 2]. Euvolemic hyponatremia requires evaluation for syndrome of inappropriate antidiuretic hormone (SIADH) but may also be the initial presentation of hypoadrenalism and/or hypothyroidism. We present a rare case of an older adult who presented with severe hyponatremia, subsequently diagnosed with panhypopituitarism. Although deficiencies involving multiple axes are common, panhypopituitarism is rare, described in 2% of primary empty sella (PES) cases [3]. This endocrine abnormality along with radiological evidence of PES led to a diagnosis of PES syndrome (PESS).

## 2. Case Presentation

A 71-year-old normotensive male, 149 cm in height (<1^st^ percentile for height) and 56.3 kg in weight (BMI: 25), with an extensive smoking history, was referred to the hospital by his primary care physician (PCP) for severe hyponatremia (117 mMol/L). His past medical records showed normal sodium levels in multiple PCP visits in the previous two years. He denied any active complaints or neurological symptoms, and initial physical examination showed an euvolemic male with feminine features and short stature. Vital signs were significant for blood pressure of 131/70 mmHg. Laboratory values also revealed mild metabolic acidosis (HCO_3_^−^: 19) and normal potassium levels, BUN of 13.1 mg/dl, creatinine of 1.27 mg/dl, and GFR of 68 ml/min/1.73 m^2^.

In the initial steps of evaluation of hyponatremia, low serum osmolality of 245 mOsm/kg but a normal urine osmolality of 309 mOsm/kg and inappropriately high urine sodium of 68 mEq/L were highly suggestive of SIADH. Due to his long-standing smoking history, we obtained a CT chest with contrast which conclusively ruled out lung malignancy as cause for ectopic SIADH.

Our suspicion for SIADH prompted us to further rule out thyroid and cortisol hormone deficiencies. Low morning serum cortisol levels (2 mcg/dl) followed by suboptimal cortisol response to cosyntropin stimulation test (injection of 1 mcg at 0 minutes, followed by cortisol measurements −8 mcg/dl at 30 minutes and 9 mcg/dl at 60 minutes) were noted. These findings coupled with a low morning serum adrenocorticotropic hormone (ACTH) level were suggestive of secondary hypocortisolism. Low free T4 with inappropriately normal TSH heralded the diagnosis of coexisting central hypothyroidism. Due to the finding of dual hormone deficiency, the diagnosis of panhypopituitarism was entertained.

**Figure 1:**
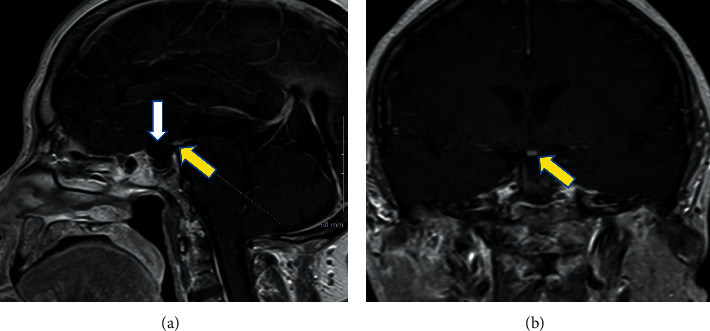
MRI brain with contrast showing empty sella with complete absence of anterior and posterior pituitary glands/infundibulum and tissue representing an ectopic hypothalamic-pituitary gland. (a) Sagittal T1 section. (b) Coronal T1 section (white arrow shows the empty sella, and yellow arrows depict the ectopic pituitary gland).

LH, FSH, IGF, and testosterone levels were all low, indicating GH and gonadotropin deficiencies. Prolactin level was mildly elevated (Table 1). To complete the workup for panhypopituitarism, an MRI brain with contrast was obtained. It ruled out pituitary adenoma or stalk compression but revealed complete empty sella (complete absence of anterior/posterior pituitary glands and pituitary stalk) and an ectopic pituitary gland in the posterior hypothalamus (Figures 1(a) and 1(b)).

At that point, we approached the patient again to get in-depth history about any prior endocrine disorders. He did acknowledge that he had required growth hormone supplementation in his teenage years as a treatment for his short stature. However, he discontinued it in a few months when he noticed no improvement. Since his teens, he was lost to follow-up by endocrinology and thus had not received any hormone supplementation. Furthermore, his underlying endocrine problems had never manifested with such severe hyponatremia during his lifetime up until this encounter.

Levothyroxine and hydrocortisone supplementation was initiated with improvement in serum sodium to 130 mMol/l noted after three days. The patient was discharged to follow-up with PCP where labs after a week showed normal sodium levels. He continues to be healthy at subsequent PCP visits.

## 3. Discussion and Conclusion

Hyponatremia with the findings of euvolemia, decreased serum osmolality, normal to high urine osmolality (>100 mOsm/kg), paradoxical natriuresis (*U*_Na_ >40 mMol/L), and absence of adrenal and thyroid disorders characterizes SIADH [4]. However, a normal acid-base balance is considered part of the SIADH criteria but is often overlooked. Occasionally, SIADH may present as metabolic alkalosis [5]. However, the patient in our case had mild metabolic acidosis. We attributed this to type 4 renal tubular acidosis which is commonly seen in ACTH deficiency [6]. Thus, in retrospect, the metabolic acidosis also provided an important clue to an underlying endocrine pathology in our case.

The correction of hyponatremia after hydrocortisone treatment is also congruent with “endocrine SIADH.” In our patient, hyponatremia can be explained by both hypocortisolism as well as hypothyroidism. For hypocortisolism, the usual mechanism of action is related to the loss of inhibitory effect of cortisol and subsequent increase in CRH which is an ADH secretagogue [7]. For hypothyroidism, the resultant decline in cardiac output and subsequent compensation by increase in ADH levels and decrease in GFR have been proposed as possible mechanisms of action [8]. All these ultimately contribute to free water retention.

Intriguingly, our patient had low ADH levels suggesting increased tubular action or heightened sensitivity to available ADH. Low ADH levels bring to light a possible underlying hypothalamic deficiency. However, ADH levels were reported at a time when hyponatremia had already been corrected. Our patient refused further testing for hypothalamic hormones.

Our case had some common and uncommon features in terms of presenting symptoms. On the one hand, absence of symptoms and incidentally detected hyponatremia is not an uncommon presentation of chronic adrenal insufficiency in the elderly and should be considered as a differential for hyponatremia [7, 9, 10]. On the other hand, we came across only a few of the cases of PES syndrome with panhypopituitarism presenting as hyponatremia [11–14]. Interestingly, they had dramatic presentations such as mania, extrapontine myelinolysis, and adrenal crisis requiring ICU admission. This further reiterates the importance of timely diagnosis and initiation of appropriate treatment. Undiagnosed, uncorrected adrenal insufficiency could potentially result in significant morbidity and mortality, particularly during periods of stress [15].

The brain MRI findings in our patient were read as an “empty sella” which is extensively described as an incidental radiological finding [16] in 8–35% of cases. It is important to differentiate primary from secondary empty sella since management differs. Secondary causes include spontaneous necrosis in preexisting pituitary adenomas, infection, autoimmune causes, intracranial hypertension, brain surgery and/or radiotherapy, cerebral trauma, and pseudotumor cerebri [17]. No obvious secondary cause was observed in our patient, and thus, through diagnosis of exclusion, we arrived at PES. Unlike our case, PES is usually described in obese woman with hypertension, typically in the 4th and 5th decade. It is important to recognize that PES with concomitant underlying panhypopituitarism is a separate entity called “PES syndrome.” It should also be borne in mind that such patients can have concomitant mild hyperprolactinemia [18] due to alterations in dopaminergic tone.

Lastly, an ectopic pituitary is a very rare condition but is a common cause of pediatric growth hormone deficiency, the likely diagnosis for this patient [19, 20]. The short stature and feminine facial features which were first noted during our patient's teenage years likely represent early evidence of hypopituitarism as a result of PES and ectopic posterior pituitary [21]. The patient's survival into old age without depending on hormonal supplementation and the sudden reappearance of endocrine deficiencies manifesting as hyponatremia remain an odd presentation.

The appropriate workup for hyponatremia in the case led to a diagnosis of panhypopituitarism enabling us to institute appropriate therapy. Even in asymptomatic individuals found to have PES, it is recommended that they should be evaluated further for underlying hypothalamic-pituitary axis deficiencies as highlighted by our patient's incidentally detected hyponatremia.

## Figures and Tables

**Table 1:** Endocrine profile.

Endocrine profile	Serum levels
Adrenocorticotropic hormone (ACTH)	12 pg/ml
Thyroid-stimulating hormone (TSH)	1.46 mcIU/ml
Free T4	0.47 ng/dl
Growth hormone (GH)	<0.1 ng/ml
IGF binding protein 3	0.6 mg/L

Luteinizing hormone (LH)	0.8 IU/ml
Follicle-stimulating hormone (FSH)	1.4 IU/ml
Testosterone	<10 ng/dL
Prolactin	19.4 ng/ml
Baseline serum cortisol	2 mcg/dl
After cosyntropin stimulation test at 30 mins	8 mcg/dl
After cosyntropin stimulation test at 60 mins	9 mcg/dl

Antidiuretic hormone (ADH)	<0.5 pg/ml

## Data Availability

The data used to support the findings of this study are available from the corresponding author upon request.

## Abbreviations

SIADH: Syndrome of inappropriate antidiuretic hormone secretion
PES: Primary empty sella
PESS: Primary empty sella syndrome
PCP: Primary care physician
TSH: Thyroid-stimulating hormone
ACTH: Adrenocorticotropic hormone
ADH: Antidiuretic hormone
FSH: Follicle-stimulating hormone
LH: Luteinizing hormone
GH: Growth hormone
IGF: Insulin-like growth factor.
